# Iturin: A Promising Cyclic Lipopeptide with Diverse Applications

**DOI:** 10.3390/biom13101515

**Published:** 2023-10-12

**Authors:** Deepak A. Yaraguppi, Zabin K. Bagewadi, Ninganagouda R. Patil, Nitin Mantri

**Affiliations:** 1 Department of Biotechnology, KLE Technological University, Hubballi 580031, Karnataka, India; deepak.yaraguppi@kletech.ac.in; 2 Department of Physics, B. V Bhoomaraddi College of Engineering and Technology, Hubballi 580031, Karnataka, India; nr_patil@bvb.edu; 3 The Pangenomics Lab, School of Science, RMIT University, Bundoora, VIC 3083, Australia; 4 The UWA Institute of Agriculture, The University of Western Australia, Perth, WA 6009, Australia

**Keywords:** iturin, production, in silico studies, biological activity, applications

## Abstract

This comprehensive review examines iturin, a cyclic lipopeptide originating from *Bacillus subtilis* and related bacteria. These compounds are structurally diverse and possess potent inhibitory effects against plant disease-causing bacteria and fungi. Notably, Iturin A exhibits strong antifungal properties and low toxicity, making it valuable for bio-pesticides and mycosis treatment. Emerging research reveals additional capabilities, including anticancer and hemolytic features. Iturin finds applications across industries. In food, iturin as a biosurfactant serves beyond surface tension reduction, enhancing emulsions and texture. Biosurfactants are significant in soil remediation, agriculture, wound healing, and sustainability. They also show promise in Microbial Enhanced Oil Recovery (MEOR) in the petroleum industry. The pharmaceutical and cosmetic industries recognize iturin’s diverse properties, such as antibacterial, antifungal, antiviral, anticancer, and anti-obesity effects. Cosmetic applications span emulsification, anti-wrinkle, and antibacterial use. Understanding iturin’s structure, synthesis, and applications gains importance as biosurfactant and lipopeptide research advances. This review focuses on emphasizing iturin’s structural characteristics, production methods, biological effects, and applications across industries. It probes iturin’s antibacterial, antifungal potential, antiviral efficacy, and cancer treatment capabilities. It explores diverse applications in food, petroleum, pharmaceuticals, and cosmetics, considering recent developments, challenges, and prospects.

## 1. Introduction

Iturin refers to cyclic lipopeptides (CLPs) that are generated from *Bacillus subtilis* and closely related bacterial strains. These compounds are renowned for their wide-ranging inhibitory properties against bacteria and fungi that are accountable for causing plant illnesses [1,2,3]. *B. subtilis*, a bacterium with a Gram-positive stain and the capacity to generate endospores, holds considerable economic importance owing to its capability to produce primary biological surfactants such as surfactin, fenthromycin, and iturin [4]. The production of these major antifungal chemicals is primarily extracellular [5]. Iturin displays structural differences within its family members while nevertheless maintaining a shared cyclic peptide structure composed of seven α-amino acids and one β-amino acid [5]. Iturin A, a highly effective lipopeptide within the iturin family, has exhibited significant antifungal characteristics and minimal toxicity, therefore presenting itself as a promising candidate for applications in bio-pesticide development and the treatment of mycoses [6,7,8]. Recent research has additionally unveiled supplementary biological functionalities of iturin, including anticancer and hemolytic characteristics [9].

The utilization of iturin encompasses a wide range of sectors. Within the realm of the food business, iturin and other biosurfactants possess a range of functions that extend beyond the mere lowering of surface tension. These functions encompass the production of emulsions, inhibition of fat globule aggregation, and improvements in product texture and shelf life [10,11]. Biosurfactants, such as iturin, have demonstrated potential in the field of Microbial Enhanced Oil Recovery (MEOR) within the petroleum sector. These substances have exhibited the ability to enhance the recovery of oil from petroleum wells and facilitate its transit through pipelines, as evidenced by many studies [12,13,14]. In addition, modern techniques such as mass spectrometry and Raman spectroscopy have been employed by researchers to characterize biosurfactants derived from *Bacillus* species [15]. In addition to their utilization in industrial applications, biosurfactants are increasingly demonstrating their relevance in a wide range of sectors. The exploration of their potential involvement in soil cleaning, agriculture, wound healing, tissue engineering, and different formulations has been documented in previous studies [5,16,17,18,19,20,21,22,23]. Furthermore, scholars are highly interested in comprehending the environmental toxicity and biodegradability of biosurfactants in order to guarantee their sustainable utilization [23,24]. As ongoing research progresses, biosurfactants are anticipated to have a substantial influence, providing environmentally friendly and adaptable solutions across multiple industries while simultaneously promoting sustainable practices and the preservation of the environment. The pharmaceutical industry recognizes the promise of iturin due to its various qualities, including antibacterial, antifungal, antiviral, anticancer, and anti-obesity effects [25,26,27]. Moreover, the diverse range of applications of iturin in the cosmetic business, such as its use as an emulsifier, anti-wrinkle agent, and antibacterial agent, underscores its adaptability [15,16].

The significance of comprehending the structure, manufacturing, and various applications of iturin is growing as research on biosurfactants and lipopeptides progresses. The objective of this review is to present a thorough examination of iturin, with a specific emphasis on its structural characteristics, production methods, biological properties, and diverse uses across multiple domains. This review aims to investigate the comprehensive range of antibacterial and antifungal properties exhibited by iturin. Additionally, it will examine the potential of iturin as an agent for combating viral infections and treating cancer. Furthermore, this research will explore the various applications of iturin in industries such as food, petroleum, pharmaceuticals, and cosmetics. Furthermore, this study will analyze the recent progress, obstacles, and prospective outlooks about the implementation of iturin in many contexts. In this review of iturin A, a comprehensive and systematic methodology was employed. The primary resource utilized was an extensive database search, focusing on academic databases such as PubMed, Scopus, and Web of Science. The search was conducted using a carefully selected set of keywords and Boolean operators to ensure relevance and inclusivity. These keywords include “Iturin A”, “lipopeptide”, “microbial biosynthesis”, “biological control”, “structural characterization”, and “biotechnological applications”. Variations and synonyms were considered to capture a wide range of relevant articles. This search strategy aimed to retrieve a diverse set of scholarly articles, reviews, and research papers that could provide valuable insights into the properties, functions, and applications of iturin A, forming the foundation for a comprehensive and evidence-based review.

## 2. Structure and Characteristics

The iturin family consists of several important members, such as iturin A, C, D, and E, as well as various versions of mycobacterium subtilisin and bacteriomycin [17]. The compounds exhibit a shared cyclic peptide structure consisting of seven α-amino acids and one β-amino acid. However, there are differences in the length of the fatty acid chain, the location of the amino acids, and the content of the compounds [5]. Iturin A, which is mostly synthesized by *Bacillus* sp., is a highly effective lipopeptide with strong antifungal properties. It consists of a cyclic heptapeptide and an α-amino fatty acid chain with 14–17 carbon atoms [5,6]. The substance has demonstrated minimal toxicity and exhibits promise as a bio-pesticide for combating plant infections and as a therapy for mycoses, as indicated by previous studies [18]. Iturin A is biosynthesized by non-ribosomal peptide synthetases (NRPSs) via the ituD, ituA, ituB, and ituC genes. Various strains, including *B. subtilis*, *Bacillus amyloliquefaciens*, *Bacillus licheniformis*, *Bacillus thuringiensis*, and *Bacillus methyltrophicus*, have been identified as capable of producing iturin A [19,20,22,24,28].

**Figure 1:**
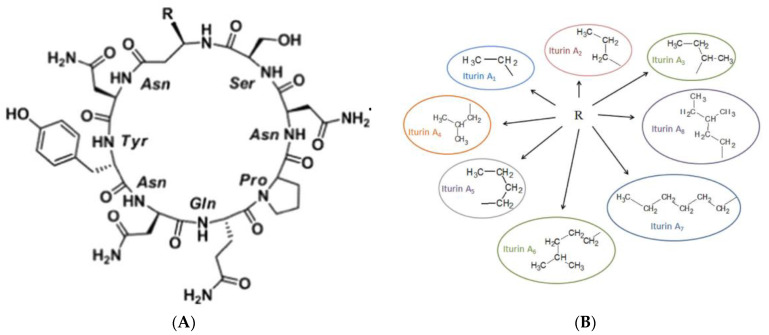
(**A**) Structure of iturin. (**B**) R: cyclic chain and homologues.

The utilization of infrared spectroscopy has facilitated the identification of β-turns within the D-lipopeptide configuration of iturin A (as depicted in Figure 1), which is synthesized by the bacterium *B. subtilis* [29]. There are eight naturally occurring isomers of iturin A, which are referred to as iturin A1 through iturin A8. These isomers have varying molecular weights, ranging from 1029 to 1084 Da [30]. Iturin A2 exhibits a type II β-turn structure, as reported in a previous study [31]. An additional investigation has revealed that iturin A2 and iturin A6 are antifungal chemicals synthesized by endophytic strains and tomato endophytes, respectively [32]. The separation and purification of iturin A are important as a result of the existence of numerous isomeric forms. Several techniques have been utilized, including the extraction of iturin A2 from the culture media of *B. subtilis* STRAIN KS03 and the subsequent determination of its chemical composition [32]. Moreover, the composition of iturin A2 comprises a type II beta-turn structure, specifically b-d-aminotetradecanoyl-l-asn2-dtyr3-d-asn4 (b-nc14nyn) and l-gln5-l-pro6-d-asn7-l-ser8. [32]. Upon decomposition of iturin A2 using electrospray ionization mass spectrometry (ESI-MS), it was shown that iturin A2 had a pronounced affinity for binding with the alkali metal ion Na^+^. The study conducted by Rautenbach, Swart, and van der Merwe in 2001 identified two distinct binding sites for non-solvent sodium ions in the two different rotation sequences of natural iturin A2. These sequences are characterized by the presence of b-amino, fourteen alkanoyl NYN, and l-gln5-l-pro6-d-asn7-l-ser8 residues. In their study, Rong et al. [33] successfully recovered the endophytic strain B21 from Osmanthus fragrans. Through structural identification, they were able to isolate and identify two antifungal chemicals, namely iturin A2 and iturin A6. Additionally, it should be noted that *B. subtilis* B47 is classified as an endophyte of the tomato plant. This particular strain of *B. subtilis* has the ability to synthesize a chemical known as iturin A2, which has antifungal properties [34]. The aforementioned results underscore the presence of distinct strains that possess the ability to generate either identical or diverse iterations of iturin A.

## 3. Production and Optimization of Iturin

### 3.1. Production of Iturin

The extraction and purification of iturin pose challenges (Table 1); however, the optimization of culture conditions can lead to larger concentrations, hence mitigating the complexity associated with extraction and purification [1]. There exist two primary methods for augmenting the production of iturin. The initial strategy entails modifying the surrounding conditions to optimize the output of the subject in question. The utilization of a solid medium in solid-state fermentation (SSF) has demonstrated notable efficacy in the synthesis of iturin A [35]. Okara, a residual product derived from soybeans with a low moisture content, is frequently employed as a substrate in solid-state fermentation processes. This utilization of okara serves the purpose of enhancing the economic worth of industrial byproducts [35]. Nevertheless, the generation of iturin A is impeded by the agitation in solid-state fermentation reactors that utilize okara, a byproduct of the tofu industry [36]. Biofilm fermentation, a process in which microorganisms aggregate and encase themselves in an extracellular matrix on a solid or liquid substrate, has demonstrated promising capabilities for the manufacture of iturin [37]. The presence of Mn^2+^ is of utmost importance in the process of biofilm formation and has been found to significantly promote the production of iturin A in the presence of other chemicals such as K^+^, Ca^2+^, cellulose, and cysteine [38].

An alternative method for enhancing iturin production involves the utilization of genetic engineering techniques. The enhancement of iturin yields can be achieved through genetic manipulation of strains at the molecular level. Previous research has demonstrated that alkaline ions, specifically potassium ions (K^+^), have the ability to enhance the rate of conversion in strains of *B. subtilis* that produce iturin [9]. The augmentation of iturin A production can be achieved by manipulating the promoters of the iturin A synthase cluster and selectively eliminating certain genes, such as AbrB, which leads to heightened expression of iturin synthase. Furthermore, previous studies have demonstrated that the incorporation of certain amino acids or precursor chemicals has the potential to enhance the production of iturin A [1].

### 3.2. Recombinant Gene Expression for Enhanced Iturin Production

The scientific community has recently shown considerable interest in the advancement of recombinant biosurfactants as they have demonstrated potential for diverse industrial applications [11]. Academic researchers are currently engaged in efforts to enhance the expression of genes responsible for biosurfactant production, with the aim of increasing yields and modifying their chemical properties. The research on the mycosubtilin and iturin A operons, as well as the lpa-14 gene, which is a homologue of the sfp gene involved in the biosynthesis of surfactin and iturin A in *B. subtilis* RB14, has become crucial [10]. An important finding in the field is the identification of a hybrid promoter, P spac, which combines the *Escherichia coli* lac operator and the promoter core region of the penicillinase gene from *B. licheniformis*. This hybrid promoter has been observed to enhance lipopeptide production by a substantial factor of five [12].

Significant progress in the field of genome shuffling techniques has led to a notable augmentation in the synthesis of iturin A. As an example, the production of iturin A, a bioactive compound, was significantly enhanced by employing a genetically modified *B. amyloliquefaciens* strain. The recombinant strain exhibited a notable increase of 2.03-fold, yielding 179.22 mg/L of iturin A in comparison to the unmodified strain [27]. In a comparable manner, it was observed that a genetically modified strain of *B. subtilis* generated a concentration of 330 µg/mL of iturin A, which represented a threefold enhancement in comparison to the production level of 110 µg/mL observed in the non-modified strain [27]. The synthesis of a putative lipopeptide biosurfactant, known as iturin A, was conducted by researchers using *Bacillus aryabhattai* as the source organism. The application of recombinant peptide engineering resulted in a notable enhancement in production yield, exhibiting a 6.7-fold rise to reach a level of 60 g/L, as compared to the unmodified strain. The biosurfactant exhibited favorable emulsification characteristics, with an E24 value of 74 ± 1.9%. Additionally, it permitted improved oil recovery, with a rate of 61.18 ± 0.85%. The unique approach presented in this study exhibits significant potential for practical applications [39]. The potential of these recombinant biosurfactants extends beyond their industrial applications. They play a vital role in the process of soil decontamination by efficiently solubilizing and mobilizing organic molecules that adhere to soil components [13]. In addition, the observed antibacterial and antiviral properties displayed by these substances indicate their potential suitability for use in biological contexts [14].

### 3.3. Optimization of Iturin Production

In order to address the challenges posed by the high costs of manufacturing and low yields, it is imperative to prioritize the optimization of lipopeptide production (Table 2). The research endeavors are centered on multiple facets, including the careful selection of strains, the enhancement of biotechnological properties through the utilization of genetic engineering techniques, the identification of optimal technical parameters, and the advancement of appropriate separation and purification procedures. The formulation of culture media is of utmost importance in facilitating the proliferation and maturation of microorganisms. The components encompassed in this context are carbon and energy sources, nitrogen sources, microelements, and supplementary nutrients such as vitamins or growth agents [40].

The influence of technological elements, namely temperature, plays a crucial role in biosynthetic processes. The selection of the working temperature is determined by finding a balance between the ideal temperature for biomass growth and the temperature at which the microbe produces the desired compound [41]. The temperature range can exhibit variability, ranging from 25 °C to 45 °C, contingent upon the specific species and strain under consideration.

## 4. Purification and Identification of Iturin

The process of purifying and identifying iturin, a lipopeptide synthesized by certain strains of *Bacillus* bacteria, involves a succession of crucial phases. Various strategies are employed in the field of lipopeptide research to accomplish thorough chemical and structural characterization. These methodologies include colorimetric assays, mass spectrometry (MS), and sequencing techniques. After the completion of the fermentation process, the bacterial cells are carefully isolated from the culture media. Following the extraction protocol, the purification of lipopeptides involves a wide range of chromatographic techniques, such as size-exclusion chromatography (SEC), ion exchange chromatography, and reversed-phase chromatography (RPC). Various methodologies, including thin-layer chromatography (TLC), ion exchange chromatography, and reversed-phase high-performance liquid chromatography (RP-HPLC), are employed for the purpose of fractionating lipopeptides, such as iturin [42]. The fractions that contain iturin are carefully collected and undergo thorough analysis using several analytical techniques, including TLC, high-performance liquid chromatography (HPLC), and MS. MS is a crucial analytical technique that plays a critical role in the identification of molecules by providing valuable insights into their molecular weight. In the realm of scientific analysis, nuclear magnetic resonance spectroscopy (NMR) has demonstrated remarkable proficiency in elucidating complex structural configurations. The identification and purification of lipopeptides, particularly in situations where there are limited numbers available, can be achieved by the use of thin TLC, a simple yet efficient approach [43]. DEAE ion exchange chromatography is commonly employed for the analysis and quantification of biosurfactants [40]. RP-HPLC is a widely recognized technique that is commonly employed for the effective separation, characterization, and purification of lipopeptides [44]. This method not only enables the evaluation of monosaccharide content and concentration, but it has also demonstrated significant value in the characterization of various lipopeptide designs [9]. In order to establish the precise identity of the purified iturin, a thorough comparison investigation is performed using existing reference data, along with comprehensive testing of its biological activity. A thorough examination of the various characteristics and possible uses of the unadulterated iturin is crucial in gaining a comprehensive understanding of its complete range of features and potential applications.

## 5. Biological Activities of Iturin

The abundance of bioactive compounds found in microbial metabolites offers a valuable reservoir of natural products that can be investigated for the development of novel pharmaceuticals. *B. subtilis*, a non-pathogenic bacterium with a wide distribution in many environmental settings, is renowned for its copious production of bioactive compounds. *B. subtilis* is extensively employed in several fields, such as food preservation, agriculture, animal husbandry, and medicine [45]. Lipopeptides produced from *B. subtilis* have been demonstrated to exhibit antibacterial, antifungal, antiviral, and various other biological properties.

### 5.1. Antimicrobial Activity

Biosurfactants exhibit a wide array of chemical compositions, such as glycolipids, lipopeptides, polysaccharide–protein complexes, phospholipids, fatty acids, and neutral lipids, which have significant surface and emulsifying properties. The ongoing emergence of bacterial resistance to pharmaceuticals necessitates the prompt identification of new antimicrobial agents, such as lipopeptides, for both therapeutic purposes and preservation [46]. The increasing demand for lipopeptides can be attributed to their potential to enhance human well-being. In the year 2003, lipopeptides were granted antibacterial authorization in the United States of America with the approval of CubicinR (Daptomycin) by the Food and Drug Administration (FDA). CubicinR is recognized as the initial cyclic lipopeptide antibiotic and is sanctioned for the management of serious blood and skin infections caused by Gram-positive pathogens [47].

*Bacillus* species are widely recognized for their significant role as efficient microbial hosts in the industrial-scale synthesis of bioactive chemicals, particularly lipopeptides. The three families of *Bacillus* lipopeptides, namely surfactin, iturin, and fengycin, demonstrate significant antagonistic effects against a wide range of phytopathogens. This characteristic renders them promising candidates for addressing challenges related to antibiotic resistance, fungal infections, and life-threatening illnesses. Lipopeptides possessing a lipid moiety of the suitable length, typically ranging from C10 to C12, demonstrate heightened bactericidal efficacy. Conversely, lipopeptides including longer carbon tails, such as 14 or 16, not only exhibit better bactericidal properties but also demonstrate improved antifungal activity. Streptomyces species, a type of actinobacteria, are recognized for their ability to synthesize diverse antibacterial lipopeptides that have use in the pharmaceutical sector.

Iturin lipopeptides exhibit significant antibacterial efficacy against a wide range of bacteria, encompassing both Gram-positive and Gram-negative strains [48]. The mechanism by which they exert their effects involves the disruption of bacterial cell membranes, resulting in the lysis of the cells [49]. The prospective uses of iturin-based antimicrobial agents in different industries, including healthcare, agriculture, and food preservation, are attributed to their efficacy [9]. These agents are effective in treating bacterial infections, managing plant diseases, and avoiding spoiling [50]. CLPs, which are produced by the activity of NRPSs, constitute a distinct category of medicinal compounds that exhibit a wide range of clinical uses. The cyclic lipopeptide families that have been subject to the most comprehensive research include surfactin, fengycin, iturin, and kurstakin [51]. Iturin is a lipopeptide characterized by a relatively low molecular mass of approximately 1.1 kDa. It consists of a peptide component composed of seven amino acid residues, together with a hydrophobic tail consisting of 11–12 carbon atoms. The amphiphilic properties of the substance indicate that its primary mechanism of action takes place at cellular membranes. Lipopeptides have attracted significant attention owing to their notable biological and physicochemical characteristics, rendering them valuable in several industries such as food, oil, and pharmaceuticals. All strains of *B. subtilis* are capable of synthesizing the iturin family of lipopeptides. The operon responsible for the production of iturin in *Bacillus* sp. is around 38–40 kilobases in length and has four open reading frames, specifically ItuA, ItuB, ItuC, and ItuD [19].

### 5.2. Antiviral Activity

The efficacy of surfactin in combating various viruses, such as Semliki Forest virus, herpes simplex virus (HSV), suid herpes virus, vesicular stomatitis virus, simian immunodeficiency virus, feline calicivirus, and murine encephalomyocarditis virus, has been demonstrated through its antiviral properties. In contrast to non-enclosed viruses, the efficacy in inhibiting the replication of enveloped viruses, specifically herpesviruses and retroviruses, is significantly higher. This observation provides support for the concept that the antiviral activity of surfactin mostly stems from its physical interaction with the external surface of the lipid bilayer, which constitutes the membrane of the virus. This interaction induces changes in permeability and, at higher concentrations, leads to the disruption of the viral membrane system as a consequence of the detergent-like properties. The researchers Shekunov, Zlodeeva, and their team investigated the potential of iturin A, a cyclic lipopeptide, as a membrane fusion inhibitor targeting SARS-CoV-2 [52]. Iturin A effectively inhibited the impact of calcium on the fusion process of negatively charged DOPC/DOPG/CHOL liposomes and other membranes, produced by PEG-8000 and SARS-CoV-2 fusion peptides.

### 5.3. Antifungal Activity

The iturin compounds have been observed to possess potent antifungal properties against a range of pathogenic fungi. Fungal growth and reproduction can be impeded through disruption of the fungal cell membrane, interference with membrane processes, and the induction of apoptosis [53]. Antifungal compounds derived from iturin exhibit significant potential for utilization in agricultural contexts to safeguard crops against fungal pathogens, as well as in medical domains for the treatment of fungal illnesses [51]. Iturin compounds exhibit a wide range of antifungal activity, effectively targeting many fungal strains, including both commonly encountered and drug-resistant ones. The authors have demonstrated efficacy against many microorganisms, including *Candida albicans*, *Aspergillus fumigatus*, *Cryptococcus neoformans*, and dermatophytes such as *Trichophyton* spp. [54]. Synergistic effects are observed when these substances are employed in conjunction with other antifungal drugs, such as azoles or polyenes [55,56]. The concurrent administration of several therapies has the potential to augment the effectiveness of antifungal treatment and mitigate the emergence of drug resistance [57]. Iturin compounds exhibit notable antifungal properties and demonstrate potential as antifungal drugs in many fields, such as agriculture, medicine, and personal care [58]. Additional research and development efforts are required to enhance the formulation of these substances, assess their safety characteristics, and fully investigate their efficacy in addressing fungal diseases and safeguarding the health and welfare of humans, animals, and plants.

The study conducted by Lei et al. [57] in 2019 examined the inhibitory effects of iturin derived from *B. subtilis* on *C. albicans*, both in vitro and in vivo. The administration of existing clinical medications for the inhibition of *C. albicans* has been linked to notable adverse effects in human subjects. The findings of the study demonstrated that iturin fractions derived from *B. subtilis* exhibited significant inhibitory effects on *C. albicans*, both in their planktonic form (with a minimum inhibitory concentration of 32 μg/mL) and inside a natural biofilm, as shown in vitro. Moreover, iturin had a potent inhibitory effect on *Candida albicans* in laboratory conditions and efficiently treated *C. albicans* infection in live organisms without inducing notable adverse reactions when administered alongside Amphotericin B (AmB). The study underscored the considerable potential of iturin as a novel pharmaceutical agent for substituting AmB in the management of profound *C. albicans* infections. Iturin showed superior efficacy in suppressing *C. albicans* while demonstrating less toxicity in comparison to AmB. Furthermore, the co-administration of iturin in conjunction with a low concentration of AmB demonstrated the ability to effectively suppress the growth of *C. albicans* colonies in a murine model of deep infection.

Extensive research has been conducted on the antifungal properties of iturin A. The substance exhibits robust antibiotic properties with a wide-ranging antifungal range, so it presents itself as a viable candidate for employment as a biological control agent in agricultural practices with the aim of minimizing reliance on chemical pesticides. Iturin A initiates a gradual alteration in the permeability of yeast cells, resulting in the liberation of potassium ions (K^+^) and essential components, finally resulting in cellular demise. The antifungal activity is ascribed to the interaction between iturin lipopeptides and the cytoplasmic membrane of the target cells, leading to an elevation in K^+^ permeability [59].

In a study investigating the effects of iturin A on postharvest fungal infections, iturin A exhibited inhibitory efficacy. The iturin-positive control exhibited the most potent inhibition [60]. The antagonistic mechanism of iturin A against *Fusarium graminearum*, a highly destructive plant pathogen, was explored by Gong et al. [61]. Iturin A exhibited noteworthy inhibitory activity, resulting in the structural deterioration of conidia and hyphae. The antifungal action of pure iturin A from strain Z-14 against *Gaeumannomyces graminis* var. *tritici* (Ggt), the soil-borne fungus responsible for wheat take-all disease, was investigated by Xiao et al. [62]. The target mechanism of iturinic lipopeptide on defense-related genes against *Colletotrichum acutatum* in pepper was revealed by Park et al. [63]. The study demonstrated that treatment with iturin CLPs resulted in the upregulation of defense-related genes. These findings emphasize the potential of iturin A as a strong antifungal drug with diverse uses in the fields of agriculture and disease management.

### 5.4. Biocontrol Agents

Biological control agents have the potential to serve as effective tools in agricultural settings, wherein they can safeguard crops against illnesses, diminish the dependence on chemical pesticides, and foster the adoption of sustainable farming methodologies [64]. Through the manifestation of antagonistic activity against a diverse array of plant pathogenic fungi, these substances impede the proliferation and maturation of fungi by causing disruptions to their cellular membranes and interfering with their essential physiological mechanisms [65]. Biocontrol agents that are based on iturin have the ability to effectively suppress a range of fungal diseases, including those that are caused by pathogens found in the soil. These agents are particularly useful in controlling diseases such as root rot, damping off, and wilt, which are caused by fungi such as Fusarium, Rhizoctonia, and Pythium species. By effectively controlling these pathogenic fungi, the impact of diseases on plants can be reduced, ultimately leading to enhanced crop productivity [51]. The literature has documented that iturin chemicals possess the ability to induce the plant’s inherent defense systems. The use of these substances has the potential to stimulate the synthesis of defense-related chemicals, bolster plant resistance against infections, and improve general plant well-being. Moreover, they present a viable ecological substitute for chemical fungicides [66]. These substances are produced from natural sources and exhibit a safety profile that is considered favorable, with minimal threats to human health, animal well-being, and environmental integrity. The utilization of iturin-based biocontrol agents can be effectively integrated with various control strategies, including cultural practices, biological control agents, and resistant crop types [67]. The implementation of integrated techniques has the potential to augment the effectiveness of disease management measures and offer a holistic resolution for safeguarding plants. The combination of these methods results in a synergistic action of iturin compounds, which effectively inhibit the growth and development of fungal infections. This biocontrol approach seems to be an efficient strategy for mitigating plant illnesses [68]. The multi-action of these compounds is directed towards several facets of fungal physiology and pathology, rendering them highly advantageous in the context of sustainable crop protection measures [69].

### 5.5. In Vitro and In Vivo Studies

The lipopeptide known as iturin, which is generated from the bacterium *B. subtilis*, has attracted considerable interest due to its possible applications. The inhibitory effects of iturin A on the hepatocellular carcinoma (HCC) cell line, HepG2, were investigated in both in vitro and in vivo settings in a study conducted by Zhao et al. [70] in 2021. Iturin A has been shown to possess the capability to infiltrate HCC cells and interfere with the cell growth cycle, resulting in a reduction in cell proliferation via mechanisms involving apoptosis, autophagy, and paraptosis. It is noteworthy that iturin A demonstrated a strong inhibitory effect on tumor growth in an HCC xenograft model while exhibiting few adverse effects. Moreover, the study revealed that iturin A exhibited the ability to reduce the expression of TGF-β1 and PD-L1. This finding suggests that iturin A may have the potential to enhance the immunological microenvironment of tumors and hinder immune evasion in HCC. Significantly, iturin A demonstrated a more prominent inhibitory impact on hepatocellular carcinoma (HCC) xenografts in comparison to prior investigations conducted on breast cancer [71], necessitating a lower dose for achieving effectiveness.

The investigation carried out by Dey et al. [72] in 2015 examined the potential anticancer properties of iturin A, a compound sourced from marine bacteria, on human breast cancer cells. This evaluation encompassed both in vitro experiments and animal models, with a specific focus on the Akt pathway as the target. The inhibitory effect of iturin A on the proliferation of breast cancer cells was assessed using MTT assays. Several drugs, including Perifosine, Erufosine, KP372-1, Triciribine, GSK690693, and MK2206, were investigated for their potential anticancer effects on the Akt pathway [73]. According to the study conducted by Dey et al. [71], it was observed that iturin A, which is obtained from marine bacteria, exhibited significant inhibitory effects on the Akt signaling network. Consequently, this inhibition led to the induction of apoptosis in breast cancer cells. Additionally, it has been observed that iturin A exhibits the capacity to impede the progression of tumors in a breast cancer xenograft model. This finding provides further evidence to suggest that iturin A holds promise as a potential therapeutic intervention for breast cancer cases characterized by heightened *Akt* levels.

Previous studies, including animal trials, have repeatedly demonstrated that the oral administration of iturin is both safe and advantageous. This administration method has been found to result in reduced levels of blood cholesterol and an enhanced presence of probiotics within the gut flora [74,75]. In 2016, a comprehensive review of the therapeutic potential and safety of iturin A was undertaken by Dey et al. [72], encompassing both in vitro and in vivo models. The enhanced anticancer effectiveness of iturin A was found to be linked with elevated DNA fragmentation and the alteration of several proteins, such as CD-31, Ki-67, p-Akt, p28 MAPK, apoptotic proteins, and anti-apoptotic proteins. A safety assessment was conducted by means of a 28-day repeated dosage toxicity and biodistribution investigation in Sprague Dawley rats. The hematological parameters of the treatment and recovery groups were observed to be unaltered as compared to the control group.

## 6. In Silico Studies of Iturin

The limited computational modeling and simulation study undertaken on iturin compounds can be attributed to the intricate nature of their structure [11,76]. However, the field of computational drug discovery has emerged as a highly effective method for understanding the process of drug design at the molecular scale. Extensive research efforts have been devoted to the advancement of pharmaceutical compounds that are specifically designed to demonstrate improved selectivity towards target proteins, hence offering potential as viable therapeutic agents [77]. Computational methodologies, such as in silico approaches and virtual screening, provide significant insights into the relationship between the structural attributes and pharmacological properties of therapeutic agents. The aforementioned methodologies are considered to be a valuable supplement to in vitro research, as indicated by previous studies [78,79].

While it is true that in silico techniques have certain limits when it comes to dealing with large molecule-based pharmaceuticals like peptides, peptidomimetics, and proteinogenic medicines [80], it is important to acknowledge that these molecules provide potential for the advancement of more precise and focused therapeutic compounds. This is due to their possession of a diverse array of functional groups that can be modified to fit the binding site of the target protein [81]. Computational methods and in silico methodologies are employed to introduce intricate compounds into the drug discovery pipeline. Molecular docking methodologies are utilized to enhance comprehension at the molecular scale and expedite the advancement of potent pharmaceutical agents [82].

The genomic analysis of *B. aryabhattai* has revealed the presence of potentially valuable lipopeptides, particularly iturin A. The findings from molecular docking and dynamics tests have provided evidence that iturin A exhibits a significant degree of binding affinity toward (1,3)-β-D-glucan synthase originating from *Candida albicans*. The presence of a stable ligand structure during several simulations suggests that *B. aryabhattai* has the capability to produce biosurfactants that are of significance to the biopharmaceutical sector [83]. A molecular docking investigation was performed on iturin A, a lipopeptide produced from *B. aryabhattai*, known for its antibacterial properties, in order to investigate its interaction with MurA. The stability of the binding was determined by the utilization of molecular dynamics simulations. The results of the investigation indicate that iturin A demonstrates a strong inhibitory impact on MurA, and the synthesis of the cell wall in *Salmonella typhimurium* relies on the functionality of the MurA protein [84]. Considerable attention has been garnered by the antibacterial, antifungal, and anticancer properties exhibited by iturin compounds. These lipopeptide compounds are synthesized by strains of *B. subtilis* [85].

The application of computer modeling and simulation methods has been essential in advancing our comprehension of the structural properties, molecular interactions, and mechanisms of action pertaining to iturin compounds. A range of computational approaches, such as molecular dynamics simulations and quantum mechanical calculations, have been employed to predict and improve the three-dimensional structures of iturin compounds [86]. These analyses provide vital insights into the conformational dynamics, stability, and interactions of iturin molecules, hence enhancing our understanding of their biological functions. Molecular docking and virtual screening approaches have been utilized by researchers to investigate the binding interactions between iturin medicines and their molecular targets [87]. The utilization of simulation tools enables the identification of potential binding sites, assessment of binding affinity, and clarification of molecular recognition mechanisms [88,89]. The degradation of cell membranes is attributed to the antibacterial properties of iturin lipopeptides [90]. The utilization of coarse-grained molecular dynamics simulations has been employed to examine the interactions between iturin compounds and lipid bilayers. The simulations conducted in this study have yielded valuable insights into the underlying mechanism of membrane disruption, particularly with regard to the creation of holes or ion channels. Additionally, the influence of structural differences in iturin compounds on their membrane-related properties has been examined [91,92].

Computational research has played a crucial role in the advancement and improvement in medications derived from iturin. The application of structure-activity relationship (SAR) analyses, virtual screening techniques, and molecular dynamics simulations plays a crucial role in identifying structural motifs and modifications that contribute to the improvement in bioactivity, stability, and pharmacokinetic properties of iturin compounds [93]. Furthermore, the incorporation of toxicity prediction and safety evaluation methodologies aids in the forecast of the potential toxicity and safety attributes of iturin compounds [94]. The application of quantitative structure-activity relationship (QSAR) models, molecular docking techniques, and in silico ADME (absorption, distribution, metabolism, and excretion) predictions provides significant insights into the potential adverse effects, metabolic fate, and pharmacokinetic properties of pharmaceuticals derived from iturin [10,95]. The utilization of computational modeling and simulation has significantly contributed to the understanding of iturin compounds, encompassing their mechanisms of action and potential applications in the domains of medicine, agriculture, and biotechnology [95]. Computational approaches play a crucial role in complementing experimental research by providing useful insights and facilitating the creation and design of treatments based on iturin.

## 7. Applications

### 7.1. Surfactants and Emulsifiers

**Figure 2:**
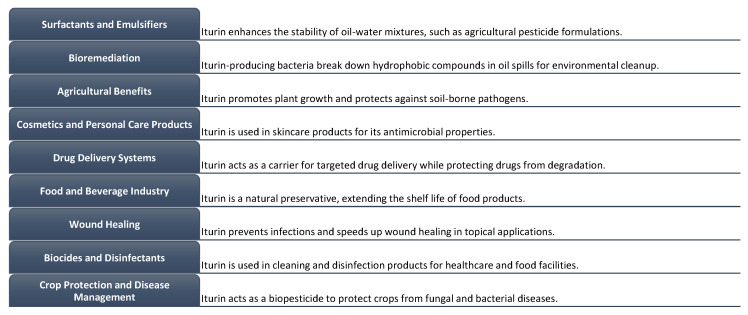
Applications of iturin.

Iturin lipopeptides exhibit surface-active characteristics, rendering them very efficient in their capacity as surfactants and emulsifiers [96]. These molecules have the ability to reduce surface tension, provide stability to emulsions, and improve the solubility and dispersion of hydrophobic compounds (Figure 2). Iturin A surfactants are chemical compounds that possess the ability to reduce the interfacial tension between two immiscible liquids or between a liquid and a solid substrate, hence facilitating their homogenous mixing [97]. Emulsifiers, conversely, serve the purpose of stabilizing and preserving the dispersion of liquids that are incapable of mixing, such as oil and water [98]. Iturin A, which is synthesized by specific strains of *Bacillus*, demonstrates robust surface-active characteristics, rendering it a very efficient surfactant and emulsifier. The compound possesses a peptide ring structure that exhibits hydrophilic properties, indicating an affinity for water, while its fatty acid tail demonstrates hydrophobic characteristics, implying a tendency to reject water [99]. The distinctive molecular structure of iturin A enables it to effectively interact with both aqueous and oily phases, hence promoting the creation of stable emulsions.

Iturin A exhibits emulsifying properties, facilitating the dispersion and stabilization of oil droplets in a water phase or vice versa. The substance is capable of arranging itself into either a monolayer or a bilayer when positioned at the boundary between two phases. This arrangement serves to decrease the tension at the interface and hinder the merging or separation of the liquids that are not mutually soluble [100]. The versatile nature of iturin A renders it advantageous for application in diverse sectors, including but not limited to food, pharmaceuticals, cosmetics, and agriculture, wherein emulsions are frequently employed. Iturin A has been found to provide potential benefits in various applications, including medication delivery. Specifically, it has demonstrated the ability to enhance the solubility and bioavailability of medicines that exhibit low solubility [101]. Iturin-derived surfactants are utilized in several industries, including cosmetics, detergents, and oil recovery, where the presence of surface-active agents is necessary.

### 7.2. Bioremediation

Iturin compounds and other biosurfactants produced by microorganisms have shown potential in environmental bioremediation [102]. Their surfactant properties enable them to solubilize and enhance the bioavailability of hydrophobic pollutants (Figure 2), aiding in the removal or degradation of contaminants in soil and water environments [103]. Iturin compounds, particularly iturin A, have been found to have significant hydrocarbon-degrading capabilities [104]. They can break down and metabolize hydrocarbon pollutants, such as petroleum hydrocarbons and polycyclic aromatic hydrocarbons (PAHs), which are commonly found in oil spills and industrial pollution. Iturin compounds can enhance the biodegradation of organic pollutants by facilitating the growth and activity of indigenous microbial populations [105]. They can act as growth-promoting factors for hydrocarbon-degrading microorganisms, stimulating their metabolic activity and increasing the degradation rates of contaminants [106]. Iturin forms complexes with metal ions, reducing their bioavailability and potential toxicity. This property can help mitigate the harmful effects of heavy metal pollution and aid in their removal from the environment. Iturin compounds contribute to soil restoration efforts by promoting soil health and fertility. They can improve soil structure, enhance nutrient availability, and stimulate the growth of beneficial soil microorganisms. This aids in the remediation of contaminated soils by facilitating the breakdown and detoxification of pollutants and promoting the recovery of the soil ecosystem [107]. It is important to note that the effectiveness of iturin compounds in bioremediation processes may vary depending on the specific contaminants, environmental conditions, and microbial communities present.

### 7.3. Agricultural Benefits

Previous studies have documented the ability of iturin chemicals to promote plant growth and bolster plant defense mechanisms. Seed germination can be facilitated, root development can be enhanced, and plant immune responses can be activated by them. The utilization of iturin-based formulations exhibits promise in their application as biofertilizers or agents that promote plant growth. This has the potential to make significant contributions to sustainable agricultural practices and enhance crop yield (Figure 2). According to the cited source [99], these substances support the growth of plants through the stimulation of root development, enhancement of nutrient absorption, and improvement in overall plant vitality. The solubilization of mineral compounds and enhancement of nutrient uptake by plants are key mechanisms via which they contribute to the increased availability of nutrients in the soil [108]. This phenomenon has the potential to result in enhanced agricultural output and increased plant efficiency. Iturin compounds exhibit potent antifungal activities and demonstrate efficacy in the management of several plant infections, encompassing fungal diseases. The utilization of iturin compounds has been shown to effectively manage fungal infections, hence mitigating potential yield losses and enhancing the general health of crops. Iturin has demonstrated the capacity to enhance plants’ resilience to abiotic stressors, including but not limited to drought, salt, and severe temperatures. Plant water balance is regulated by these molecules, which also enhance the stability of cellular membranes and trigger the activation of stress-responsive genes. This enables plants to endure unfavorable environmental conditions [109]. The utilization of iturin compounds in agricultural practices has the potential to foster environmentally sustainable farming systems by diminishing the dependence on synthetic pesticides and fertilizers. This approach can effectively mitigate the adverse effects on ecosystems and human health, as highlighted in reference [110].

### 7.4. Cosmetics and Personal Care Products

Lipopeptides, derived from iturin, exhibit surfactant characteristics and have potential use in the field of cosmetics and personal care goods [111]. Emulsifiers, stabilizers, and foam-enhancing compounds find utility in diverse formulations such as creams, lotions, and shampoos (Figure 2). Due to its potent antimicrobial activity against a range of pathogens, encompassing both bacteria and fungus, the integration of iturin A into cosmetic compositions holds potential for impeding the proliferation of germs that contribute to skin infections, acne, and other dermatological concerns [112]. This phenomenon plays a significant role in the conservation and durability of cosmetic products, while simultaneously fostering the well-being of the skin [113]. Iturin exhibits surfactant characteristics, facilitating its ability to efficiently eliminate dirt, excessive oil, and pollutants from both the skin and hair. These substances function as mild cleaners, facilitating a comprehensive and gentle cleansing process while preserving crucial moisture [114]. Cleansers containing iturin, a type of lipopeptide, have been found to offer potential advantages for persons who have oily or acne-prone skin. The antioxidant activities of the iturin compounds have been demonstrated, indicating their potential to counteract the harmful effects of free radicals and safeguard the skin against oxidative harm [115]. The observed antioxidant activity has the potential to contribute to the amelioration of ageing processes and enhance the overall aesthetic qualities of the skin [116]. According to a study conducted by researchers, iturin has demonstrated potential applications in skin conditioning, hydration promotion, and the maintenance of skin elasticity [117]. The appeal of iturin chemicals to consumers seeking ecological and sustainable cosmetic products lies in their derivation from natural sources, specifically certain *Bacillus* species. Additional investigation and formulation refinement are vital in order to ascertain the most advantageous concentrations, stability, and compatibility of iturin when employed in cosmetics and personal care merchandise. Prior to commercial implementation, it is imperative to take into account safety evaluations and regulatory factors.

### 7.5. Drug-Delivery Systems

The potential of iturin-based lipopeptides as drug-delivery vehicles has been the subject of investigation (Figure 2). The attractiveness of these substances lies in their amphiphilic properties and their capacity to spontaneously form micelles or vesicles, which enables them to encapsulate and transport hydrophobic medication [118]. The utilization of iturin in drug delivery systems can be altered by including targeting ligands or antibodies, enabling precise and directed administration to intended locations [119]. This phenomenon has the potential to promote medication accumulation at the intended site, reduce off-target effects, and improve treatment outcomes. The release rate of the encapsulated medication can be customized to achieve prolonged and regulated drug release, hence enhancing therapeutic effectiveness and decreasing the frequency of dosage through the manipulation of the delivery system’s composition and properties. The suitability of iturin for incorporation into drug delivery systems is evidenced by its reduced propensity to elicit unpleasant reactions or create long-term accumulation inside the human body [120]. The utilization of these supplementary therapeutic effects has the potential to augment the effectiveness of the administered medication, specifically in the management of infections or inflammatory disorders.

### 7.6. Food and Beverage Industry

The antibacterial capabilities of iturin compounds make them suitable for application in the food and beverage industry, specifically for the purposes of food preservation and extending shelf life [121]. The presence of these substances might impede the proliferation of bacteria that contribute to spoiling, hence preserving the overall quality and safety of food items (Figure 2). The utilization of iturin compounds as supplements in animal feed has been investigated with the aim of enhancing animal well-being and optimizing performance. The antibacterial properties of these substances have been observed to be effective against pathogens often found in the gastrointestinal system of animals. This attribute contributes to the prevention of illnesses and the improvement in feed efficiency [122]. According to a study [123], iturin compounds have demonstrated efficacy in combating foodborne pathogens, including *Salmonella*, *E. coli*, and *Listeria monocytogenes*. The application of iturin in food processing or as a component of food safety treatments has the potential to mitigate the occurrence of foodborne illnesses and improve overall food safety [124]. The amphiphilic characteristics exhibited by iturin compounds enable the encapsulation and regulated liberation of volatile flavor compounds, hence augmenting the sensory attributes and olfactory perception of diverse commodities. Flavor protection and delivery in food and beverage applications can be achieved by their utilization [125]. Additional investigation is necessary to ascertain the most suitable uses and concentrations of iturin in particular food and beverage compositions, taking into account safety evaluations and regulatory factors.

### 7.7. Wound Healing

The utilization of iturin lipopeptides has demonstrated promise in the field of wound healing. According to a study [125], these substances have antibacterial characteristics that have the potential to mitigate or manage infections in the context of wound care. The anti-inflammatory activities of iturin compounds have been documented (Figure 2). In the context of wound healing, inflammation plays a crucial role by facilitating the removal of debris and initiating the process of tissue repair [126]. The anti-inflammatory properties of iturin have the potential to assist in the regulation of excessive inflammation and facilitate a harmonized healing process. The molecule known as iturin has been proposed as a potential stimulant for the process of angiogenesis, which involves the development of new blood vessels. Sufficient blood flow is of paramount importance in the process of wound healing, as it facilitates the transportation of oxygen, nutrients, and growth elements to the specific location of the wound [127]. The potential of iturin to stimulate angiogenesis has the capacity to accelerate the creation of blood vessels, hence boosting the accessibility of essential resources for tissue repair and regeneration [50]. Furthermore, the implementation of iturin for wound healing purposes necessitates meticulous deliberation, encompassing the development of appropriate formulations, optimization of dosage, and thorough safety evaluations, prior to any potential clinical utilization.

### 7.8. Biocides and Disinfectants

The utilization of iturin compounds as biocides and disinfectants in various contexts is possible due to their antibacterial activity [50]. Microbial contamination management and hygiene maintenance can be effectively achieved by including them into cleaning solutions, surface coatings, and disinfection formulations [111]. Iturin-derived formulations have the potential to serve as effective surface disinfectants for the purpose of sanitizing and decontaminating a wide range of surfaces. These surfaces may include those found in hospitals, laboratories, food processing facilities, and public places. The antibacterial characteristics of the substance aid in the eradication of pathogenic microorganisms, hence mitigating the potential for illnesses and the spread of contaminants. Additionally, these devices have the potential to be utilized in water treatment procedures, namely for the purpose of managing microbiological contamination in water sources [128]. Iturin-based biocides have demonstrated efficacy in mitigating the growth and spread of deleterious microorganisms across various water systems, including potable water, wastewater, and industrial water. By employing these biocides, the preservation of water safety and quality can be effectively achieved.

### 7.9. Crop Protection and Disease Management

The potential of iturin-based formulations in crop protection and disease control has been the subject of investigation [57]. Biopesticides and biofungicides have the potential to serve as viable alternatives to synthetic chemical pesticides in the agricultural sector, effectively managing pests and diseases while also being environmentally beneficial [107]. It has been observed that iturin compounds possess the ability to stimulate systemic resistance in plants, hence augmenting their inherent defense mechanisms against pathogens [129]. When subjected to iturin treatment, plants exhibit the ability to trigger many defense mechanisms, including the synthesis of antibacterial substances and reinforcement of cellular barriers, enhancing their resistance against infections. iturin-based techniques have the potential to mitigate crop losses resulting from pathogenic infections through the appropriate management of plant diseases [66]. This phenomenon has the potential to enhance crop output and quality, hence guaranteeing increased productivity and economic gains for agricultural practitioners [130].

Iturin compounds exhibit a diverse array of potential applications across multiple industries, encompassing healthcare, agriculture, environmental remediation, and other sectors. In order to properly comprehend the benefits of these advancements, it is imperative to take into account practical considerations and undertake further study, optimization, and validation prior to their extensive implementation across many areas.

## 8. Conclusions

In conclusion, iturin compounds have shown extraordinary versatility, making them critically useful in many different fields. These chemicals have been shown to have antibacterial and antifungal properties, making them useful therapeutic options for a wide range of microbial infections. Because of their potential benefits in crop protection against diseases and reducing the need for chemical pesticides, the biocontrol capabilities of iturin compounds have been intensively studied in the agricultural sector. Their ability to stimulate plant growth and strengthen defensive mechanisms has made them crucial to the advancement of environmentally friendly farming methods. Because of their anti-tumor properties, iturin compounds show promise in oncology as potential targeted medications for cancer treatment. Iturin’s surfactant and emulsifying properties have also found use in the cosmetics, detergents, and oil recovery sectors. They are also used in bioremediation processes to hasten the breakdown of hydrophobic pollutants in both terrestrial and aquatic environments. Furthermore, there has been much study into the potential use of iturin compounds in areas like wound healing, biocides, disinfectants, crop protection, and disease management.

Iturin compound structural features, molecular interactions, and modes of action have all greatly benefited from the use of computational modeling and simulation research. We now have a better understanding of the biological roles of iturin molecules thanks to the important insights gained from the studies into their conformational dynamics, stability, and interactions. Several characteristics of iturin compounds have been investigated with the help of computational methods like molecular docking, virtual screening, and molecular dynamics simulations. These methods have allowed us to investigate binding interactions, pinpoint possible targets, examine membrane contacts, and assess the mechanisms underlying pore formation. In addition, the study of structure-activity relationships, predictions of toxicity, and safety assessments employed in these studies have made significant contributions to the development and improvement in pharmaceuticals based on iturin compounds. Potential side effects, metabolic fate, and pharmacokinetic features of iturin-based drugs have all been predicted with the help of computational modeling.

While studies using computational modeling and simulation have shed light on the potential of iturin compounds, more work is needed to fully realize this. The optimization of iturin-based formulations, evaluation of safety profiles, and consideration of regulatory issues are all required for its effective usage in a wide range of applications. Iturin-based medicines will continue to be conceptualized and developed with the guidance of computational approaches alongside experimental inquiries, facilitating their use in medicine, agriculture, and biotechnology.

## Figures and Tables

**Table 1:** Strategies and mechanisms used to enhance iturin production by *Bacillus* sp.

Strategy Used	Factors Evaluated	Strain	Biosurfactant Nature
RSM, OFAT one factor at a time	Carbon source (glucose, fructose, sucrose, xylose, rhamnose, and soluble starch), nitrogen source (NH_4_Cl, C_6_H_17_N_3_O_7_, urea, peptone, and soybean meal), and metal ions (ZN^2+^, Fe^3+^, Mg^2+^, Mn^2+^, Ca^2+^, and K^+^).	*Bacillus* sp. BH072	Iturin A [28]
Solid-state fermentation (SSF)	Rice bran husk, sunflower oil, coconut oil cake, cotton oil cake, corn cob, orange peel, jackfruit peel, sugarcane leaf, pineapple peel, banana leaf, cheese whey, dry yeast cells, pongamia seed cake, jatropha seed cake ground oil cake, and glucose with MSM.	*B. amyloliquefaciens*	Iturin A [36]
Mutagenesis-induced enhanced yield	Random mutagenesis using gamma irradiation.	*B. subtilis* UTB1	Iturin A [9]
Genome shuffling	Genome shuffling and gene (fenA) expression. Mutagenesis (UV, nitrosoguanidine, atmospheric, and room-temperature plasma).	*B. amyloliquefaciens* LZ-5	Iturin A [27]

**Table 2:** Lipopeptide yield for the optimum culture media composition.

Producing Microorganism	Carbon Source	Nitrogen Source	Lipopeptide Yield
*B. subtilis* MO-01	Sucrose (22.431 g/L)	Ammonium chloride (2.781 g/L)	1712 mg/L [40]
*B. subtilis* MTCC 2423	Glucose (1.098 g/L)	Yeast extract (0.426 g/L)	1501 mg/L [40]
*B. subtilis* SPB1	Glucose (40 g/L)	Urea (5 g/L)	720 mg/L [40]
*Bacillus natto* NT-6	Glucose (10 g/L)	L-monosodium glutamate (5 g/L)	563.20 mg/L [40]
*Bacillus circulans* MTCC 8281	Glucose (32 g/L)	Urea (1 g/L)	4350 mg/L [40]
